# List of Cases Received under the Care of Dr. John B. Davis, the Physician of the Universal Dispensary for Children, St. Andrew's Hill, Doctors' Commons, London; from July the 31st, 1817, to the 31st of October Following

**Published:** 1817-12

**Authors:** 


*List of Cases received under the Care of Dr.* JOHN B. DAVIS, *the Physician of the Universal Dispensary for Children, St. Andrew's Hill, Doctors' Commons, London; from July the* 31*st*, 1817, *to the* 31*st of October following.*

| DISEASES. | Number of Cases in each Month. | | | |
|---|---|---|---|---|
| | August | Sept. | October | Total |
| Febris | 7 | 2 | 5 | 14 |
| —— Remittens | 8 | 7 | 8 | 23 |
| —— Tertiana | 1 | — | 1 | 2 |
| Febricula | 6 | 3 | 1 | 10 |
| Pneumonia | 2 | 2 | 6 | 10 |
| Dysenteria | 6 | 4 | 2 | 12 |
| Diarrhœa | 12 | 10 | 4 | 26 |
| Cholera Morbus | 8 | 10 | 3 | 21 |
| Morbilli | 8 | 4 | 6 | 18 |
| Pertussis | 9 | 4 | 7 | 20 |
| Erysipelas | 7 | 16 | 3 | 26 |
| Tormina | 3 | 1 | 2 | 6 |
| Phrenitis | — | 4 | 6 | 10 |
| Convulsiones | 5 | 3 | 2 | 10 |
| Pemphigus | 1 | — | 1 | 2 |
| Icterus | 4 | 5 | 3 | 12 |
| Tabes Mesenterica | 6 | 2 | 4 | 12 |
| Roseola | 2 | 1 | 3 | 6 |
| Tussis | 3 | 1 | 2 | 6 |
| Tussis cum Febre | 2 | 3 | 1 | 6 |
| Dyspnœa | 2 | 1 | — | 3 |
| Febris Catarrhalis | 3 | 1 | 5 | 9 |
| Pleurodynia | 2 | — | — | 2 |
| Hæmoptoe | 2 | 1 | 2 | 5 |
| Eruptiones Variæ | 6 | 3 | 1 | 10 |
| Erythema | 4 | 2 | 5 | 11 |
| Cynanche Parotidœa | 4 | 1 | 1 | 6 |
| ———— Tonsillaris | 4 | 2 | 4 | 10 |
| ———— Trachealis | 1 | 2 | 2 | 5 |
| Epilepsia | 2 | — | — | 2 |
| Scorbutus | 2 | 2 | 1 | 5 |
| Vomitiones | 3 | 4 | 2 | 9 |
| Varicella | 1 | — | 2 | 3 |
| Carried over - | 136 | 101 | 95 | 332 |

| DISEASES. | Number of Cases in each Month. | | | |
|---|---|---|---|---|
| | August | Sept. | October | Total |
| Brought over - | 136 | 101 | 95 | 332 |
| Rachitis | 3 | 2 | 4 | 9 |
| Dyspepsia | 10 | 16 | 2 | 28 |
| Hemiplegia | 2 | — | — | 2 |
| Paraplegia | 1 | — | — | 1 |
| Atrophia | 3 | 1 | 2 | 6 |
| Epistaxis | 1 | 1 | — | 2 |
| Hydrocephalus Chron. | 5 | 3 | 2 | 10 |
| ——— Acutus | 3 | 1 | 3 | 7 |
| Scarlatina | 1 | 2 | 4 | 7 |
| Chorea Sancti Viti | 5 | 2 | — | 7 |
| Vermes Ascarides | 4 | 2 | 1 | 7 |
| ——— Lumbrici | 2 | 1 | 1 | 4 |
| ——— Tænia | 2 | — | — | 2 |
| Hæmatemesis | 1 | 2 | — | 3 |
| Urticaria | 5 | 3 | 2 | 10 |
| Psoriasis | 2 | — | — | 2 |
| Lepra | 1 | — | — | 1 |
| Morbus Lienis | 1 | — | — | 1 |
| Surgical Cases | — | — | — | 60 |
| Totals - | 188 | 137 | 116 | 501 |

Total number of admissions from the opening of the Dispensary, June 24, 1816, to Oct. 31, 1817 - 2497

| | |
|---|---|
| Total cured - - - - - - - - - - - - - - | 1799 |
| Total died - - - - - - - - - - - - - - - | 46 |
| Total inoculated for Cow-pox - - - - - - - | 91 |
| Upon the books, and under cure - - - - - - | 561 |
| | 2497 |

| | |
|---|---|
| Total number of Patients admitted under the Physician - - - - - - - - - - - - - - | 2115 |
| Ditto under the Surgeons - - - - - - - - | 382 |
| | 2497 |

Erysipelas was a prevalent disorder in the month of August; it principally attacked the head, face, and eye-lids. In several children at the breast, an erysipelatous tumour appeared, indiscriminately, first on one part of the scalp, and then on another; from whence it extended to the forehead, then the eye-lids and the face. The cases of erysipelas which occurred this month were numerous, and also more distinctly marked than the Reporter ever recollects to have seen.

Mumps, cholera morbus, and an inflammatory affection, similar to the inflammatory affection of the brain, in the preceding months, were frequent diseases in August. Several cases of hydrocephalus chronicus, connected with rickets and extreme debility of the digestive organs, which had been of some standing in the Dispensary, gave way to tonics, consisting of tartarized iron and columba, and the weekly interposition of purgatives of scammony and the submuriate of mercury. The health of the children labouring under this disorder, has thus greatly improved. From having had repeated attacks of convulsions, and from having been comatose with strabismus, four children out of eight, are now, at the expiration of two months from their first admission into the Dispensary, perfectly free from any convulsive tendency and coma, and are, in all respects, steadily advancing to recovery. The other four promise also to do well by a similar treatment. Two of this number have had a return of strabismus and coma, but they have been lately attacked with diarrhœa, which appears to have carried off the former symptoms.

In the month of August, September, and October, a great many cases of fever occurred, of a bilious remitting nature at their commencement. The symptoms were high pyrexia, a furred tongue with a yellow streak in the middle; vomiting; yellowness of the skin, and tunica conjunctiva; pain in the head; quick respiration; oppression and pain at the præcordia; a rapid pulse; scanty urine of a reddish colour. For three or four days, all these symptoms continued, but abated and increased repeatedly in the course of twenty-four hours: then, the patient passed gradually from this into another state, the fever changing from its first character, to fever of a typhoid nature. The tongue became hard, brown, and dry; the lips and teeth were covered with a brown crust; pyrexia increased; the pulse was more rapid; and the eye glassy. In this stage of the complaint there was delirium, with considerable pain in the head; and at times a green or yellowish fluid was vomited up. The children in whom this modification of fever appeared, were between seven and twelve years of age. In all respects, their situation was now similar to the situation of adults who were at first attacked with typhus. The fever ran on for fourteen days, and sometimes longer, before it showed any tendency to imperfect crisis—imperfect, because the solution of it did not take place by sensible diaphoresis, by evacuations from the bowels, or by epistaxis. In an almost imperceptible manner, pyrexia abated, and then the tongue grew cleaner, whiter on the edges, became covered with a healthy moisture, and was soft and smooth to the finger. If a gentle action had been excited in the bowels, throughout all the stages of the fever, this was the change at the end of fourteen or fifteen days from its attack. Next, from a state of general restlessness, which alternated with a comatose doze and delirium, the child passed into a composed and placid state, and slept comfortably. When awake it sighed frequently, was dejected, and fretful. Even though there was this remission of symptoms in the morning, or for a few hours during the middle of the day, yet, in the evening, a severe exacerbation usually came on; the face was flushed; the skin dry and parched, and the head painful. Nor was it uncommon for a child who experienced two or three such severe exacerbations to be attacked with diarrhœa, which, far from terminating the fever, in the way of critical solution, generally protracted the disease, and proved in itself obstinate of removal.

This autumnal fever among children, in every instance, appeared to depend upon some peculiar irritation in the stomach, intestines, and liver. If the secretions of the bowels were vitiated, acrid, excessive or deficient, it was perfectly obvious, that all the symptoms of the fever were then more severe. The presence of fæculent matter in the intestines was an invariable cause of aggravation. In this case, an irritative and disordered state of the viscera not only remained, but absolutely gave a character to the disease, which made it doubtful to the Reporter whether it had not first its origin in some impediment to the performance of the functions of the stomach and intestines, from this cause. Of this he is at least certain, that if the fever had not its source here, he means in a morbid irritative state of the primæ viæ, that whatever created irritation in them, always gave a new and worse complexion to it. This was proved beyond any doubt, in the success of any treatment, more particularly directed to these viscera. If the irritative action which had been supported, if not produced, by the presence of fæculent substances in the alimentary canal, had not been moderated or subdued in the begin-ing of the fever, by the repeated administration of aperi-ents, the fever then ran out to a greater length, and sooner partook of the nature of typhus. Not until the secretions of the stomach, liver, and intestines became natural, did pyrexia and other symptoms abate. These appeared to hang upon the greater or less degree of derangement in the functions of these viscera. The healthy change in the secretions could not be the result of diminution of fever: for this continued so long as the secretions were disor-dered, but abated when the irritation of the chylopoietic viscera subsided, and when they resumed their healthy functions and secreted natural fluids. In the interval, free from the exhibition of cathartics, which consisted of pulv. jalapæ, or pulv. rhei and the submuriate of mercury, dia-phoretics, with the liquor antimonii tartarizat. et liquor ammon. acetat. were serviceable; but the effect of these was unavailing, or of temporary duration, unless the a-perient was frequently repeated. One of the most effica-cious remedies was a solution of sulphate of magnesia and tartarized antimony, in such doses as promoted a re-gular discharge from the bowels, and a slight diaphoresis. Even when the fever had taken the typhoid character, the same remedy, with a free use of diluents, exposure to cool air, and a daily change from one room to another, even from the bed to the street, were of infinite service. No child died of this fever, under such treatment, severe as were the symptoms, in many instances, and modified as the fever was with acute pains in the head and side, ten-sion and tenderness of the abdomen, vomiting, and diar-rhœa.

A fever precisely like the fever of this season, has not fallen under the Reporter's notice before, among children. It was evidently the same fever as the prevalent fever of the autumn, which has attacked so many adults in every district of the metropolis. It is no subject of surprise with him, that typhus has reigned so extensively this au-tumn; but its infrequency of late years is, in the Repor-ter's mind, both a singular and an important fact. Ex-cept in a few solitary instances, he has scarcely met with typhus for three or four years, among a numerous class of poor, in the extended districts of the London Dispensary, and in a large eastern and a southern district of the me-tropolis: on the contrary, he has seen, within a few weeks, in the districts of the Surrey and London Dispensaries, not less than sixty cases of typhus. Only in two or three patients has this fever ended fatally. Though the reigning disease of the autumn, both among children and adults, it is evidently a typhus mitior which has fallen under the Reporter's notice. Its common duration has been three weeks.

Cholera morbus and diarrhœa, with enteritis, made a formidable figure in the list of diseases of the autumn. They attacked children indiscriminately, from one year old to ten and twelve; and the younger the child the more severe were these diseases. In cholera morbus, the stools were always preceded by acute pains in the intestines, and convulsive motions of the arms and legs; and after a few days' continuance of this disease, a mucous discharge, intermixed with blood, escaped from the bowels. The whole of the time, pyrexia was high, the abdomen tense and very tender. Diarrhœa, accompanied with enteritis, was very prevalent during August and September. The cases of measles in these months were numerous, but evidently milder than those recorded in the spring and winter months. When the hooping-cough followed soon after the measles, as it did in several instances in this season, it did not prove more violent, or more difficult of treatment, than in those children who had not been previously attacked with measles. Throughout the months of August, September, and October, the general character of acute diseases among children was severe. The head and the bowels were the usual seat of inflammation. When it attacked the latter, cholera morbus and diarrhœa were frequently combined with it; water in the head, when it attacked the former. In almost every acute disease, in which pyrexia was very high, and unconnected with any local inflammation of the bowels, the head always appeared to be extensively affected. Congestion in the brain might be traced, as the root whence other symptoms arose. The deep flush upon the countenance; the glassy appearance of the eye; the inability to hold up the head; the pain experienced upon exposing the eye to a strong light, and coma, plainly implied turgescence in the vessels of the brain.

In three children only did scarlatina appear. Several were brought into the Dispensary in August and September with cynanche tonsillaris, accompanied with slight pyrexia. Erysipelas, urticaria, and roseola, as well as a variety of anomalous eruptions, which were preceded by pyrexia, were very frequent. In one instance, varicella, attacked with vomiting and high pyrexia of three days' duration.

Independently of several cases of symptomatic chorea sancti viti, from worms, many idiopathic cases of this disease occurred. In a girl of eleven years of age, there was always fever with this disease; the tongue was sordid, and an irritative action prevailed in the stomach and bowels, which sometimes occasioned vomiting, at others diarrhœa. Another case, the worst the Reporter ever saw, was in a girl thirteen years old. This child had no control whatever over her limbs; her arms and legs were in perpetual motion; she could not support the body erect; the muscles of her face were hideously distorted. Sitting or lying, she was in continual motion. At night, a paroxysm of fever always came on; during which her gesticulations were convulsive. She had been in this state several weeks before she was admitted a patient into the Dispensary: she was tall and emaciated, and had consumptive symptoms. Brisk purging once in five or six days, with submuriate of mercury and scammony, proved particularly serviceable. An abatement of the more violent motions of the arms and legs always succeeded copious evacuations from the bowels.

The following nights were more tranquil, and the pyrexia less. Diaphoretics and purgatives were exhibited for three weeks. After that time, she took the volatile tincture of valerian, the carbonate of iron and columba, with evident advantage. At the end of two months she had entirely recovered from the disease. It is important to add, that the efficacy of tonics in this, and in other instances of chorea sancti viti, was greatly promoted by the occasional interposition of an active purgative. Exposure to the open air, several hours daily, assisted in the recovery of this description of patients.

About the beginning of October, many children were brought to the Reporter with pain in the head, constipated bowels, and continued pyrexia. In all of them, the actions of the arterial system were highly excited; the pulse was full, strong, and rapid; the breathing quick; the tongue white. In some of these children, who were commonly between four and ten years of age, epistaxis occurred, and was always salutary. It lowered the actions of the system, relieved the head-ach, abated pyrexia, and was a natural cure of the disease. The Reporter had no occasion to resort to general bleedings for the removal of this description of fever; but the probability is, that it might have proved a safe and efficacious remedy. This fever, it must be remembered, had nothing of the bilious or typhoid character; it partook more of synocha.

During the month of October, a vast number of children, from five to twelve years of age, were attacked with diarrhœa; some with dysentery. It was difficult to say whether many of the cases were distinctly the former or the latter disease. They appeared to be of a mixed character: one part of the intestines was inert, and its plicæ filled with scybala, which were discharged with mucus and blood; whilst another portion, which acted with increased irritability, secreted a large quantity of watery fluid mixed with bile. Purging with castor oil every morning, or with small doses of sulphate of magnesia, and an anodyne at night, succeeded in rendering the action of the bowels more uniform and regular. Submuriate of mercury, and small doses of opium, given every night at bed-time, were extremely useful. Flannel rollers carried round the body did essential good, both by maintaining uniform warmth, and by affording support to the abdominal viscera. Some of the cases of diarrhœa were followed by ascites.

Till the latter end of September and the beginning of October, inflammatory complaints of the chest were mild and few; but when the cold easterly winds of the season sat in, then catarrhal affections and pneumonia were both equally frequent and severe. In very young children, pneumonia declared itself in the most formidable manner. Cough and pyrexia were urgent, the face œdematous, and the difficulty of breathing suddenly great. Unless copious bleedings were resorted to immediately on the attack, blisters, and two or three active purgatives, the disease proved fatal in a few days. Catarrhal affections were accompanied with high pyrexia: in them the secretion of fluid in the trachea and the pituitary membrane was so copious, as, in some instances, to create alarming irritation, and to threaten sudden suffocation. No cases of complete croup occurred within the period of this Report; but many children had a hard cough with sonorous breathing, and pyrexia. One child was opened (not a patient of the Dispensary), who died of suffocation, preceded by these symptoms, shortly after having had the measles. Mr. Benjamin Davis, of the Surrey Dispensary, conducted the dissection, and has reported, that the inferior lobes of the right and left lung were inflamed; that the tracheal membrane was also inflamed throughout its whole extent, but more particularly at its bifurcations. In the bronchiæ there was pus. The heart and pericardium, as well as the abdominal viscera, were healthy. The vessels of the pia mater were distended, and shewed an appearance of inflammation.

Most of the cases of measles had a mild character; and, except in those where purging had not been freely promoted in the beginning of the disease, they were seldom succeeded by diarrhœa. The instances of jaundice were few: the cases of slight hepatic derangement very numerous. But by far the largest proportion of chronic disorders consisted in a dyspeptic state of the stomach, in mesenteric obstruction, rickets, hectic fever, and general debility of the chylopoietic viscera. In some of these children, the scrophulous diathesis strongly predominated; but in others, a morbid habit of body appeared to have been contracted from a languid and dyspeptic state of the digestive organs. Among children of the latter description, hydrocephalus chronicus was as common as among those of a scrophulous habit. It was often removed, and often returned; absorption and effusion of water alternately taking place, as the general state of the system varied. In a boy of eight years of age, an epileptic fit came on, twice or thrice in every twenty-four hours, during a whole year. He still is dull, heavy, and indifferent to those about him: his sight is imperfect, and he is incapable of walking without assistance; appearances denote him to be scrophulous; his abdomen is distended and hard. He has constant pyrexia; and, for several weeks, has been almost in a senseless state. Epilepsy in him appears to be the result of effusion into the ventricles. The more marked the symptoms are of effusion, the more violent may the next return of the fit be expected. When the attack passes by, he is revived, as if in the struggle the effused fluid had been absorbed. The Reporter is convinced, that water has been repeatedly effused and absorbed in this boy's case. At the present time, the fits are much milder, and recur only twice or thrice in the week. This the Reporter attributes to the steel and volatile alkali which he has now taken for three weeks.

Two infants, a year old each, have been admitted into the Dispensary, completely paralyzed in the lower extremities, from a cause which cannot be discovered. The children are plump, and otherwise healthy, without the slightest indication of any disorder of the spine; neither have they met with any injury. Blisters have been applied to the lumbar vertebræ without any good effect. Three cases of hæmatemesis have occurred within a few weeks, in children of ten years old. One of the children, a delicate girl, has had several returns of this hæmorrhage. For three or four days before she vomits blood, she experiences great pain and tension in the epigastric region, and severe head-ach. Ol. Ricini and small doses of opium have been very beneficial: other medicines have been rejected. By giving ol. ricini every morning, and the eighth of a grain of opium once in six hours, and changing the air, the child has entirely recovered. Erythema with considerable pyrexia, and pyrexia with numerous eruptions of an anomalous kind, have been met with in a great many instances. Several obstinate cases of psoriasis have also been admitted.

From the preceding Report it will appear, that, during the three last months, the general character of disease among children has been severe. A bilious remitting fever, which degenerated into typhus, has been very prevalent. Affections of the head and chest, cholera morbus, diarrhœa, and dysentery, have also been very frequent and very severe. The season, upon the whole, has been extremely sickly.

Mr. Benjamin Davis, of the Surrey Dispensary, has reported to me a case of fungus hæmatodes, which fell under his observation, in a child of five years of age. The testis, he states, was extensively diseased; it measured three inches and a half in length, and five in circumference; its shape was pyriform. When the tumour increased, a cough came on, and the child died hectic. Upon opening the body, a tumour was discovered, of the size of a goose's egg, in the superior lobe of the right lung, exactly resembling the tumour of the testis. He suggests whether the disease of the lungs could have been communicated by absorption? He thinks, and with great probability, that both tumours were formed by a similar morbid action; and that the extirpation of the testicular tumour would have been of no service.

Case

